# The COVID-19 pandemic presents an opportunity to develop more sustainable health workforces

**DOI:** 10.1186/s12960-020-00529-0

**Published:** 2020-10-31

**Authors:** Ivy Lynn Bourgeault, Claudia B. Maier, Marjolein Dieleman, Jane Ball, Adrian MacKenzie, Susan Nancarrow, Gustavo Nigenda, Mohsin Sidat

**Affiliations:** 1 School of Sociological and Anthropological Studies, University of Ottawa & Lead, Canadian Health Workforce Network, Ottawa, Canada; 2 Technische Universität Berlin/European Observatory On Health Systems and Policies, Berlin, Germany; 3 Senior Advisor Royal Tropical Institute (KIT)/Associate Professor Athena Institute, VU University, Amsterdam, The Netherlands; 4 University of Southampton, Southampton, UK; 5 WHO/PAHO Collaborating Centre On Health Workforce Planning and Research, Dalhousie University, Halifax, Canada; 6 Southern Cross University, Lismore, Australia; 7 National Autonomous University of Mexico, Mexico City, Mexico; 8 University Eduardo Mondlane, Mondlane, Mozambique

## Abstract

This commentary addresses the critically important role of health workers in their countries’ more immediate responses to COVID-19 outbreaks and provides policy recommendations for more sustainable health workforces. Paradoxically, pandemic response plans in country after country, often fail to explicitly address health workforce requirements and considerations. We recommend that policy and decision-makers at the facility, regional and country-levels need to: integrate explicit health workforce requirements in pandemic response plans, appropriate to its differentiated levels of care, for the short, medium and longer term; ensure safe working conditions with personal protective equipment (PPE) for all deployed health workers including sufficient training to ensure high hygienic and safety standards; recognise the importance of protecting and promoting the psychological health and safety of all health professionals, with a special focus on workers at the point of care; take an explicit gender and social equity lens, when addressing physical and psychological health and safety, recognising that the health workforce is largely made up of women, and that limited resources lead to priority setting and unequitable access to protection; take a whole of the health workforce approach—using the full skill sets of all health workers—across public health and clinical care roles—including those along the training and retirement pipeline—and ensure adequate supervisory structures and operating procedures are in place to ensure inclusive care of high quality; react with solidarity to support regions and countries requiring more surge capacity, especially those with weak health systems and more severe HRH shortages; and acknowledge the need for transparent, flexible and situational leadership styles building on a different set of management skills.

## Introduction

It seems to have taken this pandemic for all of us to more explicitly value health workers. Health workers occupy a unique position in response to COVID-19. The epidemiology of the virus contributes to an unprecedented increase in the volume and acuity demand on the health workforce, while at the same time it diminishes health worker supply. As the backbone of health systems, health workers are the key responders to the crisis as it unfolds, and in being at the point of care, they are also most at risk.

**Fig. 1:**
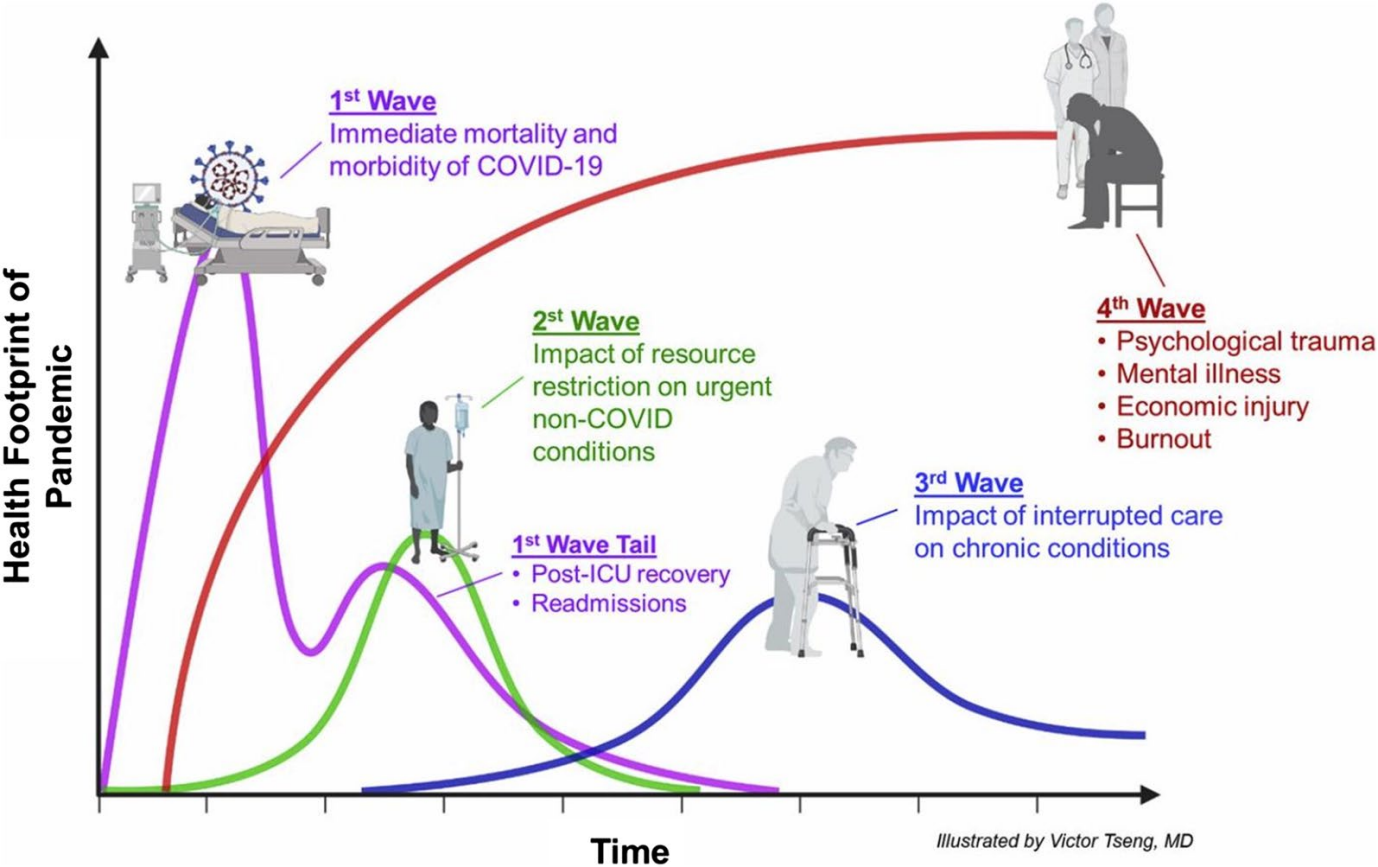
The Four waves of a pandemic, Dr. Victor Tseng; reprinted with permission

A useful depiction of the different types of the health impacts of the pandemic over the short, medium and long term is depicted in Fig. 1 [1]. This includes a first wave addressing the immediate response to COVID-19, which may also entail post-intensive care unit (ICU) recovery and readmissions. Three additional population health needs include a second non-COVID-19 wave of the backlog of other urgent health conditions. A third wave depicts the impact of interrupted care of chronic conditions, which could be in primary or long-term care settings. The backdrop to each of these waves is a fourth wave highlighting the psychological trauma and economic injury caused within the broader population. *For each of these waves the corresponding health workforce requirements that parallel each COVID-19 wave need to be considered.*

In this commentary, we describe the range of the initial health workforce considerations in response to the pandemic, highlighting some of the key issues considered and not fully considered across a number of selected countries. We begin with a discussion of how health workforces were readied for the first wave through increasing surge capacity and access to personal protective equipment (PPE). We add the growing concern with the psychological health and safety of health workers as the pandemic unfolded. Across both issues, we highlight the important equity dimensions. Next, we discuss attempts to increase health worker flexibility in how they respond to the pandemic, which raises the issue of under-recognised workforces. The cross-cutting issue of the need for open and flexible leadership is also highlighted.

## Supply and surge capacity: increase numbers

Paradoxically, pandemic response plans in country after country often failed to explicitly address the health workforce requirements and implications on the workforce itself. Initial prominent concerns were whether facilities, regions and countries would have sufficient supplies of resources to respond to the surge. Concerns about the number of ventilators, masks and other PPE can obscure whether there are sufficient operators trained to manage life sustaining equipment, and generally provide care around intensive care beds. As Professor Ivan Cavicchi at the University of Tor Vergata in Rome explains,

“*We made a mistake, especially in Lombardy. … We were totally focused on increasing the number of beds in intensive care units, without having enough anesthesiologists.*” [2]

A range of policy options have been explored and undertaken to rapidly increase workforce capacity, each with associated benefits and challenges. A cross-country analysis of policy measures by the European Observatory on Health Systems and Policies has shown that the majority of countries used a multitude of different strategies to scale up their workforces within a short time-period [3]. This included expanding staff capacity among the existing workforce, calling upon trainees and retirees, and integrating internationally educated health professionals (IEHPs). Selected examples are noted below.

### Recalling inactive health workers

Voluntarily recalling retired staff or those on leave or inactive is a common response across many countries. In the Netherlands, for example, there was an overwhelming response from 20,000 retired health workers and those on leave to come back to the health sector in response to COVID-19, with over 3000 working by the end of March [4]. In Germany, the president of the German Federal Association also called upon retired physicians to return to medical work or to help with case tracing or telephone helplines [5]. Regulatory authorities have responded quickly to enable inactive practitioners to come back to work as well as fast-track trainees (discussed below) [6]. These calls are not without concern because of the often neglected need for short-term retraining, but also because the greater potential risks to older workers of exposure to the virus [7].

### Fast-tracking trainees

Fast-tracking trainees near the end of their programs is another common strategy. In Australia, for example, nursing students were employed as assistant nurses to free up registered nursing time to deal with more acute cases. In Germany, following a call from the federal medical student association, over 20,000 medical students have registered to work in clinical practice by the end of March. In the Netherlands, medical students supported GP practices and provided health information to the general population [8]. Trainees have also volunteered, as for example in Jamaica, to help support frontline health workers by providing child care [9].

Fast-tracking of trainees requires additional considerations, such as those identified by Jane Ball in an opinion piece in the Nursing Times [10]:

*“1. Students are students—they require supervisions and support. They are not yet fully trained, nor assessed as being safe to practise independently;*

*2. If students need to become part of the workforce in an emergency, they are no longer students—but part of the workforce;*

*3. They must be free to make this choice themselves. …;*

*4. If they become part of the workforce, they must be paid appropriately relative to level of skills and have employment rights.”*

### Integrating internationally educated health professionals

Integrating IEHPs already in country is another strategy, albeit controversial. In the UK, an accelerated process was implemented to get international nurses onto the register more quickly [11]. A strategy that some countries have considered is to import foreign nurses or physicians, such as Cuban doctors proposed by some Indigenous communities in Canada to support the response to COVID-19 [12]. Policy decision-makers have been cool to these proposals [13]. For example, a recent call from a local politician in Canada to allow-more-foreign-trained-doctors-to-help-with-covid-19-crisis sparked controversy with regulatory authorities concerned with safety implications [14], but an emergency policy was enacted nonetheless on a short-term basis [15]. In Mexico, concerns were that these strategies neglect endemic local underemployment [16], and suspicions were unleashed about the functions that this personnel will be able to perform and the way the will be contracted and paid over the course of the epidemic [17].

### Redeploying health workers across jurisdictions/countries

Calls for health worker mobility have also come from more heavily affected areas. The Governor of New York State, for example, asked health workers from across US to help shore up the surge particularly in New York City, a favour later returned when there was need in the state of Utah [18]. In Canada, the province of Nova Scotia developed a “Good Neighbour Protocol” to facilitate ‘sharing’ of health workers across and within jurisdictions after H1N1 that was re-activated for COVID-19 [19]. China sent health workers to Italy at the height of its surge [20]. This solidarity is also needed for those areas that face critical workforce shortages across the globe.

## Physical safety and access to PPE

Safety concerns and access to PPE in a state of global shortage, have figured prominently in policy discussion and public discourse. Early in the crisis, the World Health Professions Alliance, representing a range of health workers, called upon governments to prioritise support for healthcare workers in the frontline against coronavirus [21]. They detailed how health workers.

*“are putting themselves at risk as they battle to protect their communities, often without the required personal protective equipment, such as masks and hazardous material suits, that can keep them safe from infection and therefore able to carry on their vital work.”*

Health workers have figured prominently in the number of those infected with COVID-19, and in COVID-19-related deaths [22, 23].

Lack of access to PPE has significantly reduced health workforce capacity, either because workers have become exposed and subsequently quarantined, or have become infected or sick, or have not come to work or walked off the job because of the risks they bear [24]. For instance, the Berlin Association of Statutory Health Insurance Physicians sent an open letter to Berlin’s mayor and the Minister of Health warning about a potential collapse of the ambulatory care system if the supply chain for PPE remained irregular [25]. In the Netherlands at the beginning of April, where 900 out of the 2500 nursing homes reported COVID-19 infections, some workers chose to quit their jobs because they lacked sufficient PPE [26].

Access to PPE has revealed invisible hierarchies within the health workforce, including those that reflect broader gender and racial dimensions. Initial calls of support for nurses and doctors failed to recognise the risks to staff in administration, transport and cleaning roles. Although we are starting to see a more sophisticated view of the different risk profiles, there were a number of instances across many countries where certain cadres of health workers were not considered in the calls for PPE [27].

The differential impact of the COVID-19 virus to racialised persons is also expressed in the disproportionate infection and death rates amongst racialised health workers [28], and how this intersects with their status in the health care hierarchy. In Canada, a memorial for health workers who have died from COVID-19 reveals a disproportionate impact on racialised workers, particularly those in the poorly recognised and protected long-term care sector [29].

Even when accessible, the gender dimension of PPE can be notable. The Gender Equity Hub of the Global Health Workforce Network, for example, pointed out how PPE can be challenging for the largely female health workforce, as it is often not designed with women’s bodies in mind [30]. Conversely, guidance from the CDC has recognised the impact of different types of men’s facial hair for safety and fit of protective masks [31]. Similar considerations were needed for health workers who wear turbans, hijabs or other religious head coverings.

## Psychological health and safety: multiple caring dilemmas

The dilemmas faced by health workers during the COVID-19 pandemic reveal another assault on their safety, this being to their psychological health and safety [32]. It has become clear that the response to COVID-19 is more akin to a marathon than a sprint, and working at or beyond full capacity is not sustainable in the long or even medium term.

Anticipatory anxiety at the preparatory phase emerged at first, which gave way to exhaustion when the epidemic curve hit exponential growth. Burnout from too much work, combined with challenging, high-pressure and morally demanding working conditions is particularly notable for those in emergency and intensive care. This includes having to make difficult decisions about who has access to ventilators and who suffers in their absence because they are considered less likely to survive [33].

Moreover, frontline workers face difficult emotional situations as they support patients in the last moments of their lives when family and relatives are not allowed to be with their family member. Wearing needed protective equipment hampered being able to provide emotional support [34]. Dilemmas also emerged when concerns about whether capacity would be sufficient to respond to the crisis outweighed guidance about when exposed health professionals should be quarantined [35].

The *caring dilemma,* to borrow a concept from Susan Reverby [36], faced by health workers concerned about potential transmission of the virus to their families when they come home from caring for patients is also emerging as a key concern. Responses have included quarantined spaces within health worker homes [37], where possible, or in local hotels or donated recreation vehicles parked in hospital parking lots [38]. These responses raise yet another caring dilemma of being away from families at the same time as they are sheltering at home with schools closed.

Providing mental (peer) support is required, and valuable lessons from current experiences can be learned for the future crisis situations [39].

## Providing care in new ways—increase flexibility

In addition to efforts to safely increase the number of providers within health worker cadres, efforts to undertake work differently, and to shift tasks and increase the flexibility of health workers across the workforce have emerged. Here is another way a whole of the health workforce approach is relevant.

### Task shifting and new skill mixing

Opportunities for workforce flexibility include the shifting or delegation of tasks and new skill mixing innovations leveraging the full scope of skills available within and outside of the health workforce. In Australia, for example, several physiotherapists were trained to work in acute respiratory teams [40]. In the UK, dental offices were asked to redeploy some of their dental staff for National Health Service work—particularly those with sedation skills; podiatric surgeons have been deployed to manage orthopaedic medical wards and assist in ‘proning’ critically ill patients. Other forms of deployment including shifting health workers freed up from cancelled elective surgeries to other sectors in greater need, a strategy adopted in Australia and Canada [41].

Flexibility in staffing ratios supports workforce flexibility. This has included guidance regarding needed variability in acute care staffing ratios to capture both ‘regular’ and ‘surge’ versions—specific to COVID-19 [42]. In Germany, for example, the Ministry of Health has suspended minimum staffing levels for nurses to allow for more flexibility on nurse placements in hospitals during the crisis [43]. Researchers in Australia have also used previously established staffing ratios to estimate numbers of additional ICU nurses and physicians that would be required to operationalise additional ICU beds and ventilators should the country be compelled to use them all [44]. Unfortunately, jurisdictions often lack even these most basic guidelines on workforce adequacy, hindering efforts to monitor and adjust these in response to the pandemic [45].

Shifting tasks may require some upskilling if the skill set is not within the existing cadre of worker. One of the specific interventions considered in Canada is to training up more Registered Nurses (RNs) to handle ventilators, in case of a shortage of Respiratory Therapists (RTs), given that there are many more RNs than RTs in the jurisdiction (of a factor of 30 to 1) [46].

### Rapid expansion of virtual care

There has been a rapid uptake of telehealth by most primary care providers, including in settings where this was previously considered infeasible. In Canada, for example, new fee codes for virtual care were fast-tracked immediately where previously governments limited these types of funding options [47, 48]. In Germany, the 2018 law on telemedicine, which was previously slowly taken up in practice, has seen a rapid adoption during COVID-19 [49]. Virtual care also affords opportunities for new models of care, including point-of-care testing and for patients to go directly to specimen collection points (not requiring a GP referral). This complemented the return of retirees as telehealth was seen as a way for older clinicians to more safely deliver care. In the US, retirees were brought back in telehealth roles to reduce their risk exposure [50].

## Taking a broader view of health workforces

Renewed interest in workers in public health roles is emerging after years of chronic underfunding and understaffing [51]. Several countries have taken measures to expand the public health workforce, employing more public health specialists and workers to help with contact tracing, staffing help lines and organising quarantine measures [52]. In Canada, the province of British Columbia announced it would invest $1.6 billion to hire 7000 health care workers to support a major influenza vaccination campaign to prevent the combination of COVID-19 and influenza from overwhelming it [53].

Hardest hit from the pandemic has been the older adult, or long-term care sector. The vulnerability of the residents was mirrored in the vulnerabilities of their care workers, a situation that predated the pandemic, but for which it brought to the fore of public attention through the daily briefings of COVID-19 infections and deaths [29]. In Canada, where over 80% of deaths are linked to long-term care, a recent national opinion poll revealed over 90% of the public consider a systemic review of long-term care facilities, notably in terms of management and level of staffing, to be paramount [54].

## Leadership and decision-making

Across these different strategies adopted and adapted, change management in the short, medium and longer terms bears consideration. In the short term, management structures and systems require distributed leadership ensuring strong governance if and when managers become exposed or fall ill. Different types of situational leadership skills are also needed—beyond the clinical—to manage the crisis at the point of care, including flexible, empathetic and open communication [55]. Leadership will also be needed to recognise how this crisis reinforced existing inequalities in the health workforce, as well as in care for the broader population [56]. Workers across disciplines at the frontline need to be recognised in decision-making. This means creating space and opportunities to have management positions occupied by women, people from ethnic minorities, various religious backgrounds where there are clearly identified leadership gaps.

Leadership at the political level is also significant. The politicisation of COVID-19 and the response to it carries the risk of hampering equity and both technical and allocative efficiency, directly risking the safety of the whole of the health workforce. It is clear that a whole of country approach as well as international cooperation are required to allocate scarce resources in a timely way to where they are needed, as well as accommodate for localised adaptation according to demographic profiles.

The responses to this pandemic, and in particular the considerations of health workforce implications, has also revealed the importance of different political, health systems and cultural approaches to creating and deploying surge capacity. Systems with central government coordination, led by policy-makers who are open to scientific evidence-based recommendations, have been better able to respond in a more coordinated way. At the same time, care has to be taken to avoid cumbersome administrative procedures than can hamper decision-making for changes in care provision that are required immediately. There is a delicate balance between a central command and control model vs. one that allows for more local authority and decision-making.

## Future planning considerations

This pandemic has clearly changed how health workers provide care, are deployed and managed. It has also laid bare the consequences of the lack of attention to health workforce considerations and how necessarily these are for improved pandemic preparedness and responsiveness. This lack of attention has been notable for workforces in particularly neglected sectors, such as in long-term care and public health.

What will be the legacies of these pandemic responses? Will there be better planning, including health workforce planning? Will it be like the Canadian response to Severe Acute Respiratory Syndrome (SARS) in 2003, where an initial flurry of policy and concerns with public health workforce responsiveness, some of which quickly subsided without full implementation? It is important to resist the tendencies towards stasis in the system when crises are overcome.

Future pandemic preparedness plans should routinely include processes for estimating health workforce requirements based on projections of the pandemic spread, and incorporate options for rapidly scaling up the health workforce through modelling and scenario planning. Sufficient financial resources to ensure these scenarios can be implemented rapidly and at scale will be necessary. Pandemic preparedness requires readily available and flexible back up options for surge capacity. Many health systems which have moved towards a lean management approach of the health workforce will need to reconsider in light of how this strategy significantly limits surge capacity in times of crisis.

## Conclusion

While support and appreciation is seen for all those health workers putting themselves at risk to save other people’s lives and to provide care in extremely difficult circumstances across many societies, more explicit policies and practices to support health workers are needed for a sustainable health workforce through the pandemic and further into the future. As we move from the initial ‘shock’ of the pandemic, the health workforce adaptions and practices required to sustain workers will be longer term than first envisaged. The second and subsequent waves of the pandemic, arising from changing lock-down and social distancing measures, will create an echo of the four waves of both population health needs depicted in Fig. 1 and consequent health workforce requirements. Countries will need to move beyond crisis management and better integrate the full range of health workforce considerations into subsequent planning phases, and rigorously monitor and evaluate the health workforce response along with its overall response to a pandemic.

## Data Availability

Not applicable.

## Abbreviations

GPs: General practitioners
ICU: Intensive care unit
IEHPs: Internationally educated health professionals
PPE: Personal protective equipment
RNs: Registered nurses
RTs: Respiratory therapists
SARS: Severe acute respiratory syndrome
